# Dimethylacetamide alone or in combination with glycerol can be used for cryopreservation of ovine semen

**DOI:** 10.1590/1984-3143-AR2020-0036

**Published:** 2021-01-14

**Authors:** Gabriel Felipe Oliveira de Menezes, Rodrigo Freitas Bittencourt, Fernando de Lima Cardoso, Maicon Pereira Lents, Elisiane Sateles dos Santos, Renata Oliveira Barreto, Edivânia Oliveira de Jesus, Mónica Madrigal Valverde, Antonio de Lisboa Ribeiro

**Affiliations:** 1 Escola de Medicina Veterinária e Zootecnia, Universidade Federal da Bahia, Av. Adhemar de Barros, 500, Salvador, Bahia, 40170-110, Brazil.; 2 Universidad de Costa Rica 1501-2060, Cuidad Universitaria Rodrigo Facio, San José, Costa Rica.

**Keywords:** semen, cryoprotectants, artificial insemination, sheep

## Abstract

Dimethylacetamide has been included in different extenders for the cryopreservation of semen from species with promising results. The objective of this study was to evaluate the use of dimethylacetamide (DMA) in different concentrations, associated or not with glycerol (GLY), for the cryopreservation of ovine semen, and its effects on *in vitro* sperm parameters and post-thaw *in vivo* fertility. Five semen samples of five adult Santa Ines sheep (n=25) were used. The collected ejaculates were divided among the seven treatments for subsequent cryopreservation. The treatments presented different concentrations of DMA and GLY, being divided as G1: GLY 6%; G2: DMA 3%; G3: GLY 5% + DMA 1%; G4: GLY 4% + DMA 2%; G5: GLY 3% + DMA 3%; G6: GLY 2% + DMA 4%; G7: GLY 1% + DMA 5%. %. Post-thawing of the straws, aliquots were evaluated for computerized sperm kinetics (CASA) and plasma membrane integrity, using fluorescent probes and flow cytometry. After the *in vitro* evaluation of the sperm parameters, *in vivo* testing was performed by laparoscopic artificial insemination of 72 females. The post-thaw total motility (%) evaluated by CASA were 51.4, 51.4, 50.1, 53.6, 52.3, 52.8 and 46.9, respectively, for the seven groups. And the plasma membrane integrity (%) were 19.7, 28.4, 22.3, 29.4, 24.3, 17.9 and 16.9, respectively. There were no differences (P> 0.05) between the treatments for the parameters of spermatic kinetics and membrane integrity. For females inseminated with semen from the control group (G1, GLY6%), the percentage of pregnant females was 36.1%, a result similar to that obtained with G3 treatment (GLY5% + DMA1%). In conclusion, dimethylacetamide, either alone or in combination with glycerol, can be used for cryopreservation of ovine semen.

## 1. Introduction

Glycerol is the most widely used cryoprotectant for freezing sheep semen. However, due to its high osmolarity, glycerol causes damage to post-thawing spermatozoa. Thus, the investigation of other cryoprotectants that allow better sperm indices after thawing is justified (Alvarenga et al., 2005; Salamon and Maxwell, 2000; Okuda et al., 2007). The use of amides has been presented as an alternative to glycerol because they offer lower viscosity and molecular weight, resulting in greater permeability of the plasma membrane, which reduces the osmotic stress to spermatozoa (Alvarenga et al., 2005).

The use of dimethylacetamide (DMA) to preserve semen has been reported for several species with satisfactory results, such as in equines (Medeiros et al., 2002; Melo et al., 2007), birds (Mosca et al., 2016), fish (Varela et al., 2012; Alves et al., 2016), and swine (Bianchi et al., 2008a). In sheep, DMA was not evaluated in association with glycerol or at concentrations beyond 3 and 6% of DMA (Bittencourt et al., 2018). Similarly, no fertility study with Glycerol-DMA association in ovine was found in the literature.

Considering the need to maintain the viability of post-thawed ovine spermatozoa, interest in identifying a more efficient cryoprotectant and its ideal concentration for successful use during sperm cryopreservation has arisen. This study aimed to evaluate if DMA could either substitute integrally the glycerol or least reduce the used GLY concentration, minimizing their negative effects on sperm post-thawing and fertility. In addition, another goal was to study the possibility of a positive effect when associating sperm cryoprotectants that act thourgh different mechanisms.

## 2. Materials and Methods

### 2.1 Location and management of animals

All procedures were approved by the Committee on Ethics in the Use of Animals of the School of Veterinary Medicine and Animal Science of the Federal University of Bahia, protocol: CEUA 41/2014. The experiment was carried out in different locations according to the research steps, between April 2018 and November 2018. Semen cryopreservation was performed at the experimental farm in the city of Entre Rios - Ba region with latitude 11º56'31 “south, longitude 38º05'04” west and at the Laboratory of Animal Reproduction, with latitude 13º00'16.7 “south, 38º30'31.6” west, belonging to the School of Veterinary Medicine and Animal Science of the Federal University of Bahia - UFBA. Following andrological examinations, twenty-five semen samples from five adults, clinically healthy Santa Inês rams, ranged in age from 18 to 24 months and with body condition score of 3 (on a scale of 1-5) and maintained on *Brachiaria decumbens* grass supplemented with species-specific mineral salt were used. An *ad libitum* water supply was provided.

### 2.2 Semen collection and evaluation prior to freezing

Semen was harvested using an artificial vagina and a female as a dummy. After two consecutive semen collections, volume, color, and turbidity were assessed visually. Total and progressive motility, vigor and turbulence were assessed by using a phase-contrast microscope (×100 magnification). The ejaculates that did not conform to the recommendations of the Brazilian College of Animal Reproduction - CBRA (Henry et al., 2013) for *in natura* semen were discarded. A Neubauer chamber was used for the calculation of sperm concentrations, with semen diluted in the proportion of 10 μL to 3990 μL (1:400) of deionized water. Aliquots were also collected for the supravital test with eosin dye - EOS (Cedenho and Tokunaga, 1995).

### 2.3 Elaboration of experimental groups

The ejaculate was diluted in medium containing egg yolk-Tris (Bittencourt et al., 2008), plus two cryoprotectants: glycerol (GLY) and dimethylacetamide (DMA), alone or in combination, with a maximum concentration of 5% DMA and 6% GLY. For this, seven experimental groups were formed: G1: GLY6%; G2: DMA3%; G3: GLY5% + DMA1%; G4: GLY4% + DMA2%; G5: GLY3% + DMA3%; G6: GLY2% + DMA4%; G7: GLY1% + DMA5%. G2 was used as the control medium for the use of DMA, based on the results of previous studies conducted by our research team (Bittencourt et al., 2018).

### 2.4 Semen cooling and freezing

Thereafter, each sample was aliquoted into seven pre-warmed tubes to achieve a calculated total of 200 × 10^6^ spermatozoa with progressive motility. Then, 0.5 mL of the respective extender medium was added slowly. The total amount of spermatozoa per treatment group was sufficient for at least two insemination doses of 100 × 10^6^ spermatozoa with motility and packed in French straws (0.25 mL). Subsequently, the samples were cooled to 5 °C (0.46 °C/min) (Model 518C, Minitub do Brasil Ltda, Porto Alegre, Brazil), left to equilibrate for 2 h (Bittencourt et al., 2014), and were then frozen in liquid nitrogen vapor.

### 2.5 Evaluation of post-thaw semen

The semen samples were thawed in a water bath at 37 °C for 30. After thawing, the semen from each group was deposited in preheated microtubes (1.5 mL) and maintained at 37 °C.

Sperm kinetics were evaluated at the Animal Reproduction Laboratory of the Santa Cruz State University (UESC), using a computer-assisted sperm analysis (CASA) (Sperm Class Analyzer SCA, Microptics, SL Version 5.1, Barcelona, Spain), configured for ovine. A particle area from 3 to 70 μm^2^ was classified as slow speed, when the measured velocity was between 10 and 45 μm/s; medium speed, when velocity was between 45 and 75 μm/s, and fast speed, when velocity was over 75 μm/s; progressive, when sperm obtained more than 80% rectilinearity (STR); circular, when they obtained less than 50% linearity (LIN). The sperms with hyperactivity movement pattern (HYPERACT) were calculated when at curvilinear velocities (VCL) = 35 to 500 μm/s, STR = 85 to 100% and lateral head displacement (ALH) = 2.5 to 100 μm.

An aliquot taken from the thawed sample was diluted in phosphate-saline-PBS buffer (Cavalcante et al., 2005) to obtain a final dilution concentration of approximately 48 × 10^6^sptz/mL (Davis and Katz, 1992). After 5 to 10 min of stabilization (37 °C), 5 μL of the diluted semen (Palacín et al., 2013) was transferred to a preheated slide (37 °C) preparation and loaded for microscopic analysis (Eclipse 50i, Nikon®, Tokyo, Japan), under 100x magnification. Images from four different fields were captured through a camera (602fc, Basler AG, Ahrensburg, Germany), totaling 100 images / second / field, with a minimum count of 250 cells.

The following sperm parameters were evaluated: total (TM, %) and progressive (PM, %), curvilinear velocities (VCL, μm/s) and mean (VAP, μm/s) and straight (VSL, μm/s), lateral head displacement (ALH, μm), LIN (%), STR (%), crossed flagellar beat frequency (BCF, Hz) and HYPERACT (%).

The evaluation of plasma membrane integrity was performed at the Laboratory of Molecular Biology and Immunology of the Institute of Health Sciences - ICS of UFBA. Samples were analyzed using a Flow Cytometer (BD FACSCalibur®, BD Biosciences, Franklin Lakes, New Jersey, USA). Two fluorescent probes were used: carboxyfluorescein diacetate (DIC, 21879, Sigma Aldrich), fluorescent green, and propidium iodide (PI, P4170, Sigma Aldrich), which fluoresces in red, in a working solution containing PBS®, DIC at a final concentration of 20.0 μM and PI at the final concentration of 15 μM (Harrison and Vickers, 1990; Mendoza et al., 2012). A total of 5 μL of PI and 5 μL of DIC were added to each 200 μL sample of semen diluted in PBS, at a concentration of 2 × 10^6^ sperm / mL in each cryogenic tube. For this analysis, a count of at least 100,000 events was established. Spermatozoa were classified as non-injured (DIC + / PI-) and injured (DIC + / PI + and DIC- / PI +). The DIC- / PI- sperm subpopulation was not counted as neither “injured” nor “non-injured”.

### 2.6 Synchronization of females and artificial insemination

Seventy-two Santa Inês female sheep with clinically healthy body scores of 3 (range 1-5) belonging to the Entre Rios Experimental Farm of the Federal University of Bahia were used. These animals were kept in *Brachiária decumbens* pasture, supplemented with mineral salts specific for the species and with an *ad libitum* water supply. All animals underwent a previous clinical and gynecological evaluation, as well as following a schedule of vaccination and worming. The females were randomly divided into two experimental groups, selected by following *in vitro* evaluations, which showed no difference (P> 0.05) among the treatments; however, the treatment that reached the highest percentages (G3) in relation to the parameters of VCL, VAP, and VSL, was compared with the control group. Group 1 (control) consisted of 36 females inseminated using laparoscopy (Killen and Caffery, 1982) with frozen semen with GLY6%; Group 2 consisted of 36 females inseminated with frozen semen with GLY5% + DMA1%.

The ewes were synchronized using a short protocol, which began on day zero (D0) with the placement of an intravaginal device containing 0.33 g progesterone (CIDR®, Pfizer, Guarulhos, Brazil) which remained in place for 8 days. On D7, 300 IU of equine chorionic gonadotrophin (eCG, Novormon®, MSD Saúde Animal, São Paulo, Brazil) and 0.125 mg of sodium cloprostenol (PGF2α, Ciosin ®, MSD Saúde Animal, São Paulo, Brazil) were administered. After 34 h of P4 (D9) withdrawal, 200 IU of human chorionic gonadotropin (hCG, Chorulon ®, MSD Saúde Animal) was administered intramuscular injection (IM). On D10, approximately 52 h after removal of the intravaginal device, laparoscopic intrauterine inseminations (Killen and Caffery, 1982) were performed, according to the treatments used, with 100 x 10^6^ sperms / 0.25mL. All animals were evaluated by transrectal ultrasonography in B mode using a linear 7.5 MHz frequency transducer (Mindray DP-50 Vet, Shenzhen, China) 30 days after inseminations for confirmation of pregnancy.

### 2.7 Statistical analysis

Data analysis was performed using the Statistical Analysis System (SAS) - version 9.0 (2002). For the spermatic parameters, a descriptive analysis was first performed, with the results being expressed as the mean ± standard deviation. The normality of data distribution was assessed using the Shapiro-Wilk test. All the studied parameters presented a non-normal distribution (TM, PM, VCL, VAP, VSL, ALH, LIN, STR, BCF, HYPERACT, DIC, PI) and therefore the Kruskal-Wallis test was used to verify possible differences of the sperm parameters among the experimental groups analyzed. The gestation rates in the experimental groups were compared using a frequency dispersion study with the chi-square test (X^2^). The level of significance adopted for all analyses was 5%.

## 3. Results

There were no individual effects (P> 0.05) of rams on the spermatic results found. Analysis of sperm kinetic parameters after thawing showed no difference (P> 0.05) between treatments and the control group (Table 1). Similary, the different GLY-DMA associations promoted similar quality of the sperm post-thaw. As observed for sperm kinetics, the plasma membrane integrity parameters did not differ (P> 0.05) among cryopreserved semen samples with different concentrations of glycerol and dimethylacetamide. In the *in vivo* fertility test, we observed that body scores of the study ewes did not differ (P> 0.05) between the experimental groups. The results observed *in vitro* were repeated *in vivo*, and evaluation of frozen semen fertility after artificial insemination at fixed time by laparoscopy did not differ (P> 0.05), independent of the experimental group used. For females inseminated with semen from the control group (G1, GLY6%), the percentage of pregnant females was 36.1%, a result similar to that obtained with G3 treatment (GLY5% + DMA1%).

**Table 1:** Parameters of sperm kinetics evaluated by computer-assisted analysis (CASA) and plasma membrane integrity (%) analyzed using flow cytometer, after freezing-thawing sperm cells of Santa Ines rams, cryopreserved with different concentrations of glycerol (GLY) and dimethylacetamide (DMA).

PARAMETERS	Fresh semen	TREATMENTS						
		G1	G2	G3	G4	G5	G6	G7
**TM (%)**	85.5% ± 0.6^a^	51.4 ± 21.8^b^	51.4 ± 24.3 ^b^	50.1 ± 18.7 ^b^	53.6 ± 21.5 ^b^	52.3 ± 21.6 ^b^	52.8 ± 24.3 ^b^	46.9 ± 23.2 ^b^
**PM (%)**	-	10.2 ± 8.6	8.5 ± 5.3	8.1 ± 5.2	9.9 ± 6.0	8.9 ± 6.1	7.0 ± 5.0	6.7 ± 4.0
**VCL (µm/s)**	-	58.3 ± 15.1	51.8 ± 14.7	57.8 ± 17.5	56.2 ± 14.9	53.3 ± 14.1	49.7 ± 19.5	50.0 ± 15.8
**VAP (µm/s)**	-	37.9 ± 16.6	31.8 ± 11.8	36.8 ± 16.8	35.5 ± 12.9	31.9 ± 10.9	30.7 ± 13.8	29.4 ± 10.4
**VSL (µm/s)**	-	27.3 ± 17.5	22.4 ± 9.9	25.8 ± 16.3	25.2 ± 11.7	22.1 ± 9.9	20.9 ± 10.9	19.9 ± 7.8
**ALH (µm)**	-	3.6 ± 0.7	3.6 ± 0.9	3.5 ± 0.5	3.6 ± 0.6	3.6 ± 0.6	3.6 ± 0.4	3.7 ± 0.8
**LIN (%)**	-	43.6 ± 16.7	42.0 ± 9.2	42.0 ± 12.6	43.4 ± 12.4	40.2 ± 9.9	38.7 ± 11.5	39.4 ± 7.0
**STR (%)**	-	67.2 ± 12.5	68.8 ± 7.2	66.9 ± 9.7	68.8 ± 8.9	67.1 ± 8.0	64.8 ± 10.9	67.0 ± 5.8
**BCF (Hz)**	-	8.5 ± 1.7	8.8 ± 2.5	8.8 ± 1.4	8.9 ± 1.6	9.5 ± 1.7	9.9 ± 3.3	8.9 ± 1.9
**HYPERACT (%)**	-	6.8 ± 8.5	5.5 ± 4.4	5.4 ± 5.1	6.2 ± 5.0	5.2 ± 4.5	4.6 ± 3.6	3.9 ± 2.6
**INJURED (%)**		75.6 ± 12.9	64.9 ± 14.9	72.7 ± 10.2	65.1 ± 15.0	69.1 ± 14.9	76.0 ± 14.0	75.5 ± 15.3
**NON-INJURED (%)**		19.7 ± 11.5	28.4 ± 15.7	22.3 ± 9.1	29.4 ± 14.0	24.3 ± 14.1	17.9 ± 13.6	16.9 ± 15.3

**G1**: GLY6%; **G2**: DMA3%; **G3**: GLY5%+DMA1%; **G4**: GLY4%+DMA2%; **G5**: GLY3%+DMA3%; **G6**: GLY2%+DMA4%; **G7**: GLY1%+DMA5%. Sperm parameters in CASA: MT = Total motility, PM = progressive motility, VCL = track velocity, VAP = smoothed-path velocity, VSL = straight line velocity, ALH = amplitude of lateral head displacement, LIN = linearity, STR = straightness, BCF = beat cross frequency and HYPERACT = hyperactivity. Spermatozoa with plasma membrane integrity were classified as NON-INJURED and the sperms with plasma membrane injured were classified as INJURED, using two fluorescent probes: carboxyfluorescein diacetate (DIC) and propidium iodide (PI). Values followed by different lowercase letters on the same row differ from each other by the Kruskal-Wallis test (P< 0.05).

## 4. Discussion

Based on the results of the study, we confirm that DMA can integrally substitute the glycerol and, when they are used in association with each other, it can reduce the GLY concentration, although without providing better post-thaw sperm maintenance rates.

Amides and glycerol are penetrating cryoprotectants responsible for the cryoprotection of sperm cells, but amides have lower molecular weight than glycerol and are more permeable to the plasma membrane, as well as tend to induce less osmotic shock to the cells after thawing, which causes less damage (Alvarenga et al., 2005). However, in our study, the lowest osmotic stress expected for the groups with isolated DMA or in higher concentrations associated with glycerol was not observed. Thus, the computerized sperm kinetics parameters were similar (P> 0.05), independent of the experimental group analyzed.

The glycerol and dimethylacetamide have diferente biochemical properties and both can protect sperm cells during cryopreservation, with cryoprotectant properties achieved through different mechanisms (Bianchi et al., 2008a). This was demonstrated in this study. The GLY and DMA alone protected ram sperm by their specific mechanisms, providing similar results for maintaining sperm kinetics parameters after thawing. This corroborates the results of studies with other species (Bianchi et al., 2008a; Rosato and Iaffaldano, 2013; Seifi-Jamadi et al., 2017; Vafaei et al., 2019) that DMA proved to be a safe alternative for sperm cryopreservation with satisfactory post-thaw motility rates.

The efficiency of the association of DMA with glycerol (GLY) has previously been tested for cryopreservation of equine semen (Medeiros et al., 2002). Those authors observed that sperm kinetics measured through CASA was favored (P <0.05) when there was an association between DMA and GLY when compared to GLY alone. Similar results were obtained by Pinho et al. (2014) with boars sperm. However, differently from the reported by those authors, the positive effect promoted by the combination of the cryoprotection mechanisms of GLY and DMA was not verified with rams, which suggests that the association can exert different cryoprotective activities in the different species. It is important to consider that there are structural differences in sperm plasma membranes among species (Ladha, 1998), as well as their different interactions with the components present in the diluent, such as ovine semen which, in comparison to equines, have better post-thawing results when glycerol is used at higher concentrations (Vidament et al., 2009).

It was observed that high concentrations of DMA (6%) in the diluent cause deleterious effects on the viability of ovine sperm cells, post-thawing (Bittencourt et al., 2018). Similar results were observed using acetamide (Silva et al., 2012) in the dilution medium (3% and 5%). The authors found that this amide caused a deleterious effect on progressive motility and the integrity of the acrosomal and plasma membrane, probably due to the greater permeability of the plasma membrane and consequent high concentrations inside the cells, causing irreversible damage (Silva et al., 2012). However, no deleterious effects on sperm membrane integrity were observed with the 5% DMA concentration in the diluent. These variations between findings in the different studies can be justified because it is known that the deleterious effects of cryoprotectants on the sperm plasma membrane are also correlated with the cryopreservation method, which can lead to the destabilization of the plasma membrane in such a way that favors cytotoxic action of the cryoprotectant (Li et al., 2005). Although it is difficult to compare our results with other studies and their specific processing methodologies, in our experiment, all cryopreservation processes were similar, regardless of the treatment used. Thus, the effect of the method could not be determined; in addition, no differences (*P*> 0.05) were observed in the sperm kinetic parameters and plasma membrane integrity among groups, even when using advanced methods with high accuracy for sperm evaluation, such as the computer assisted sperm analysis (CASA) and flow cytometry, the latter evaluating on average 400 thousand events.

The evaluations used *in vitro* for seminal analysis have the objective of predicting the fertility potential of the spermatozoa *in vivo*. However, not all evaluations have a high correlation with fertility rates, and artificial insemination (AI) is essential for the confirmation of the fertile potential of semen, post-thawing (Wu et al., 2013). Thus, after artificial insemination with frozen semen in the two diluent groups (G1: GLY6% and G2: GLY5% + DMA1%), no differences (P> 0.05) were observed for the conception rates of the inseminated females. Similar findings were found in a study of boar semen, which did not differ (P> 0.05) between inseminated animals using GLY (3%) and diluent medium containing GLY (3%) + DMA (1%) (Thurston et al., 2003).

It is known that several factors can interfere in the fertility rate of inseminated sheep, among them are the body score of the females, the type of protocol used (Hill et al., 1998), the experience of the inseminator (Godfrey et al., 1999), the semen quality after thawing, and season of the year (Anel et al., 2005). In this study, these factors were controlled in both groups.

The fertility rate of this study (36.11%) was moderately lower than in the literature, with rates of 42% (Masoudi et al., 2017) and 50% (Rabassa et al., 2007). Results similar to the present work were observed after the insemination of sheep (Bittencourt et al., 2018) with frozen semen using diluents containing DMA (3%) and GLY (6%). That is the only study found in the literature with *in vivo* fertility test for ovine frozen semen with amides. And, according to those authors, the use of DMA demonstrated that it could be considered a safe alternative for cryopreservation of ram spermatozoa, providing satisfactory post-insemination fertility rates. In fact, the similarity in conception rates confirms that both DMA and GLY can protect ovine sperm cells during cryopreservation, by different mechanisms, corroborating the findings for computer-assisted sperm analysis and sperm membrane integrity by flow cytometry and according to boar repports (Bianchi et al., 2008b).

The positive effect promoted by the GLY-DMA combination, as well as *in vitro* tests, was not verified in fertility test. As we did not identify other studies that evaluated the *in vivo* fertility of cryopreserved sperm with GLY-DMA combination in sheep, future studies using different cryoprotectans combinations and concentrations are suggested.

## 5. Conclusions

Based on the results of the analysis of *in vitro* sperm parameters and *in vivo* fertility, it is concluded that either dimethylacetamide alone or in combination with glycerol, at concentrations tested, can be used for cryopreservation of ovine semen.
